# Supplementary material for: A conserved molecular switch in Class F receptors regulates receptor activation and pathway selection
Source: Nat Commun. 2019 Feb 8;10:667. doi: 10.1038/s41467-019-08630-2 (PMC6368630; doi:10.1038/s41467-019-08630-2)
Supplement: Supplementary file 1 — Supplementary Information [file 41467_2019_8630_MOESM1_ESM.pdf]

Supplementary Information

A conserved molecular switch in Class F receptors regulates receptor activation and pathway selection

Wright and Kozielewicz et al.

Supplementary Note 1

List of abbreviations:

7TM	-	seven-transmembrane-spanning
BRET	-	bioluminescence resonance energy transfer
CRD	-	cysteine-rich domain
CFP	-	cyan fluorescent protein
DVL	-	Dishevelled
ERK1/2	-	extracellular signal-regulated kinase1/2
FZD	-	Frizzled
GFP	-	green fluorescent protein
GLP-1	-	Glucagon-like peptide 1
GPCR	-	G protein-coupled receptor
H8	-	helix 8
IL1, 2, 3	-	intracellular loop 1, 2, 3
MD	-	molecular dynamics
mG	-	mini G protein
SMO	-	Smoothened
TM6/7	-	transmembrane spanning helix 6/7
WNT	-	WNT/Int-1 family of proteins

a

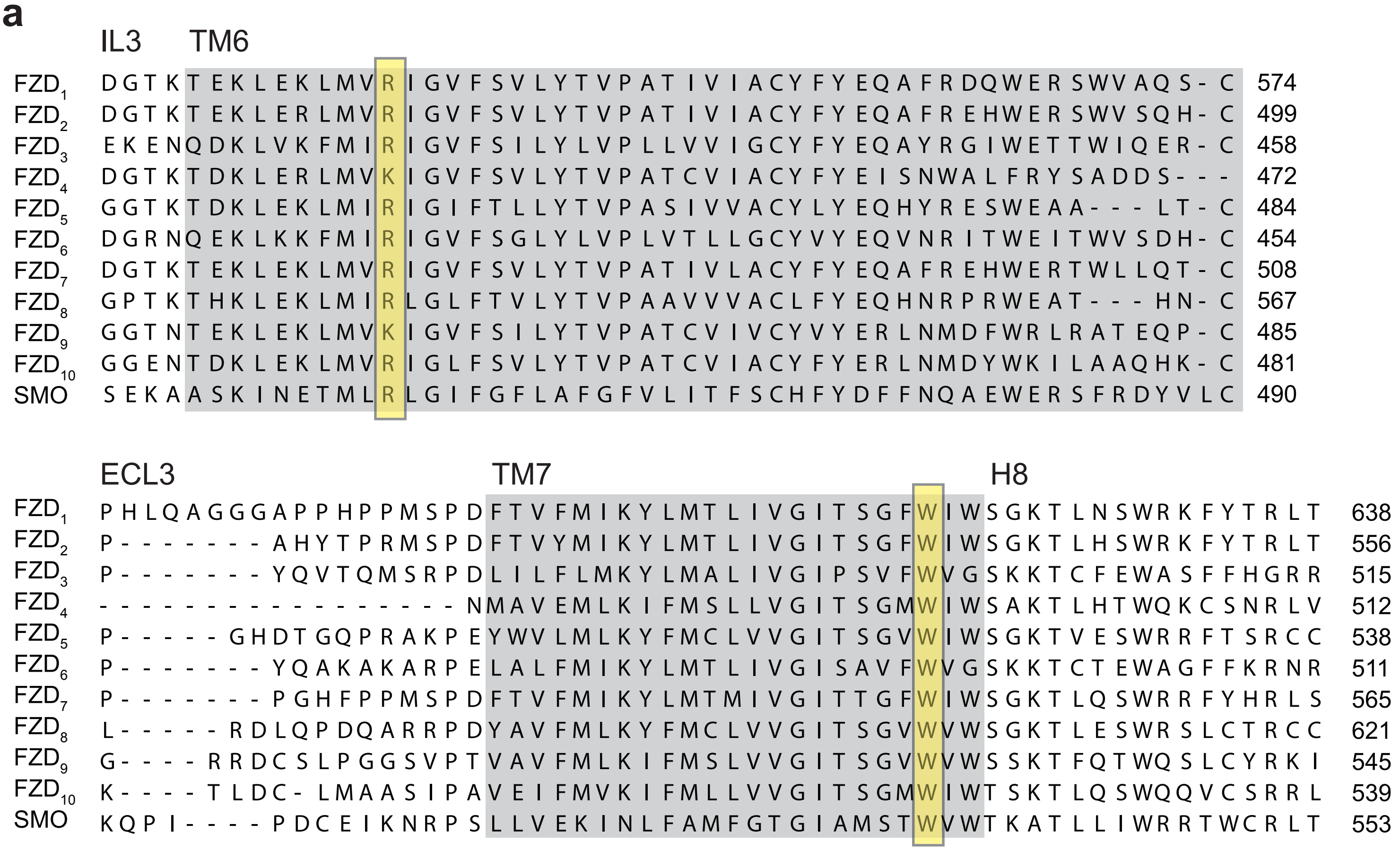
IL3
TM6
FZD1 DGTKTEKLEKLMVRIGVFSVLYTVPATIVIACYFYEQAFRDQWERSWVAQS-C 574
FZD2 DGTKTEKLERLMVRIGVFSVLYTVPATIVIACYFYEQAFREHWERSWVSQH-C 499
FZD3 EKENQDKLVKFMIRIGVFSILYLVPLLVVIGCYFYEQAYRGIWETTWIQER-C 458
FZD4 DGTKTDKLERLMVKIGVFSVLYTVPATCVIACYFYEISNWALFRYSADDS--- 472
FZD5 GGTKTDKLEKLMIRIGIFTLLYTVPASIVVACYLYEQHYRESWEAA---LT-C 484
FZD6 DGRNQEKLKKFMIRIGVFSGLYLVPLVTLLGCYVYEQVNRITWEITWVSDH-C 454
FZD7 DGTKTEKLEKLMVRIGVFSVLYTVPATIVLACYFYEQAFREHWERTWLLQT-C 508
FZD8 GPTKTHKLEKLMIRLGLFTVLYTVPAAVVVACLFYEQHNRPRWEAT---HN-C 567
FZD9 GGTNTEKLEKLMVKIGVFSILYTVPATCVIVCYVYERLNMDFWRLRATEQP-C 485
FZD10 GGENTDKLEKLMVRIGLFSVLYTVPATCVIACYFYERLNMDYWKILAAQHK-C 481
SMO SEKAASKINETMLRLGIFGFLAFGFVLITFSCHFYDFFNQAEWERSFRDYVLC 490
ECL3
TM7
H8
FZD1 PHLQAGGGAPPHPPMSPDFTVFMIKYLMTLIVGITSGFWIWSGKTLNSWRKFYTRLT 638
FZD2 P-------AHYTPRMSPDFTVYMIKYLMTLIVGITSGFWIWSGKTLHSWRKFYTRLT 556
FZD3 P-------YQVTQMSRPDLILFLMKYLMALIVGIPSVFWVGSKKTCFEWASFFHGRR 515
FZD4 ------------------NMAVEMLKIFMSLLVGITSGMWIWSAKTLHTWQKCSNRLV 512
FZD5 P-----GHDTGQPRAKPEYWVLMLKYFMCLVVGITSGVWIWSGKTVESWRRFTSRCC 538
FZD6 P-------YQAKAKARPELALFMIKYLMTLIVGISAVFWVGSKKTCTEWAGFFKRNR 511
FZD7 P-------PGHFPPMSPDFTVFMIKYLMTMIVGITTGFWIWSGKTLQSWRRFYHRLS 565
FZD8 L-----RDLQPDQARRPDYAVFMLKYFMCLVVGITSGVWVWSGKTLESWRSLCTRCC 621
FZD9 G----RRDCSLPGGSVPTVAVFMLKIFMSLVVGITSGVWVWSSKTFQTWQSLCYRKI 545
FZD10 K----TLDC-LMAASIPAVEIFMVKIFMLLVVGITSGMWIWTSKTLQSWQQVCSRRL 539
SMO KQPI----PDCEIKNRPSLLVEKINLFAMFGTGIAMSTWVWTKATLLIWRRTWCRLT 553

b

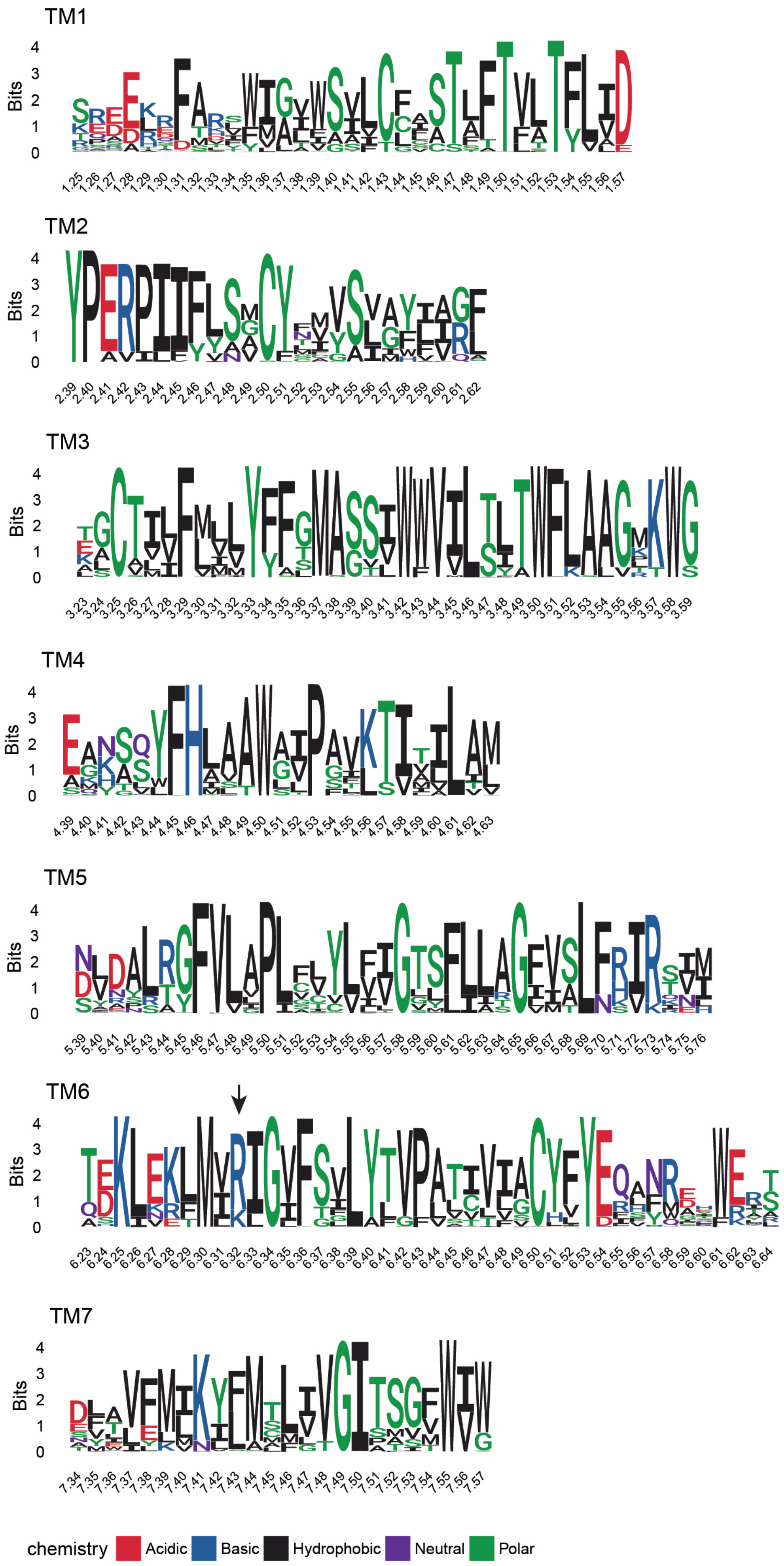
TM1
TM2
TM3
TM4
TM5
TM6
TM7
Bits
chemistry
Acidic
Basic
Hydrophobic
Neutral
Polar

Supplementary Figure 1. Phylogenetic conservation of R/K$^{6.32}$ and W$^{7.55}$ in Class F receptors. (a) Sequence alignment of human FZD and SMO paralogs, generated using the MAFFT aligner in the G-INS-i mode. The conserved R/K$^{6.32}$ (TM6) and W$^{7.55}$ (TM7) residues are highlighted with yellow boxes. (b) Sequence logos for transmembrane regions of Class F receptors. Transmembrane regions were extracted from a large-scale alignment of Class F receptors (Supplementary Information) using the homology model of FZD_6 as guide. Residues are numbered using the FZD_6 Ballesteros-Weinstein residue identifiers. Residue R/K$^{6.32}$ was marked with an arrow. Sequence logos were generated using the ggseqlogo package (*1*).

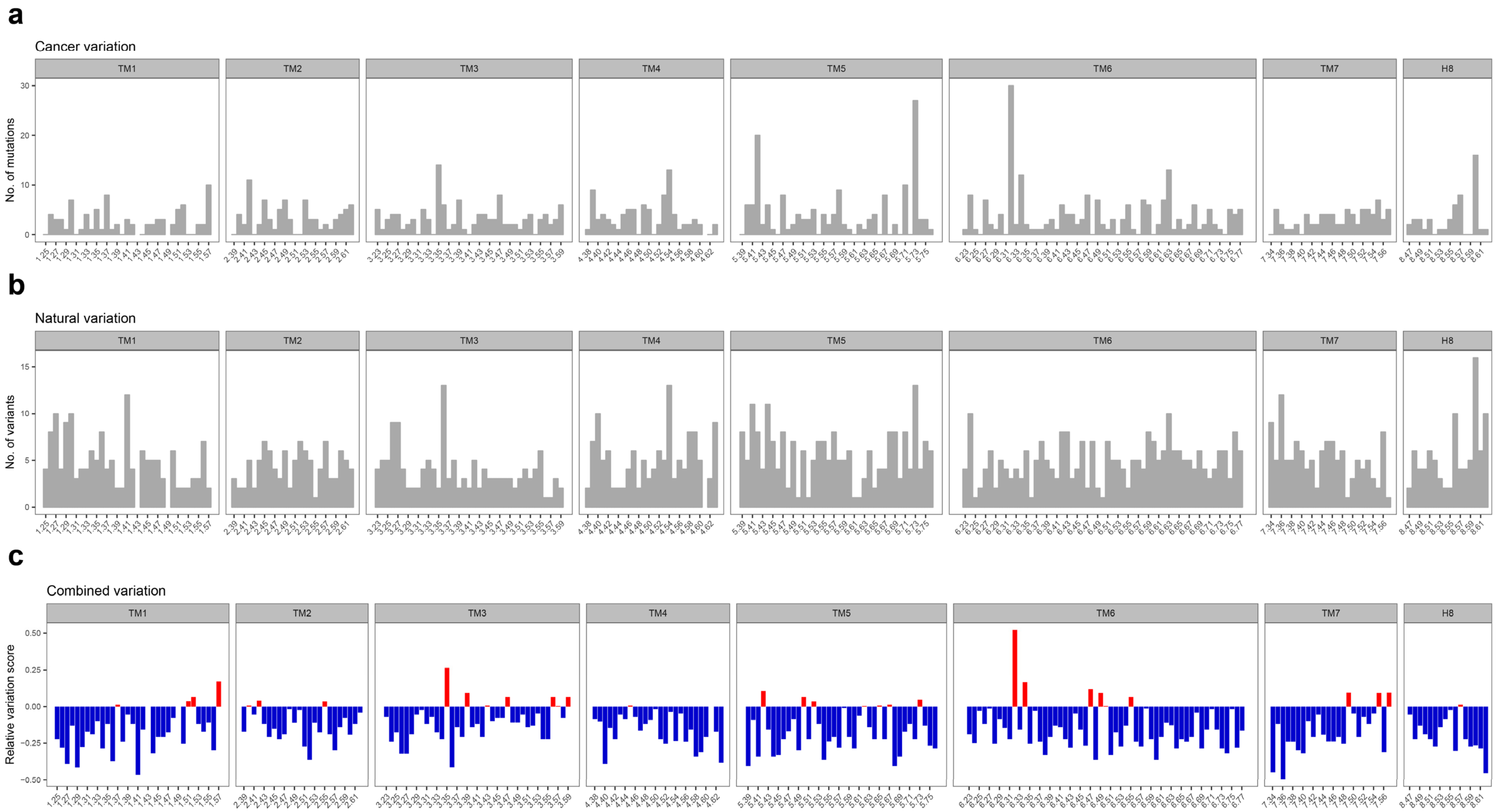

Supplementary Figure 2. Cancer and natural variation in Class F receptors. (a) Amino-acid altering variants mapping to the 7TM regions in Class F receptors obtained from the gnomAD database. (b) Cancer mutations mapping to the 7TM regions in Class F receptors obtained from the cBioPortal database. (c) Sitewise relative variation score in the 7TM regions of Class F receptors.

a

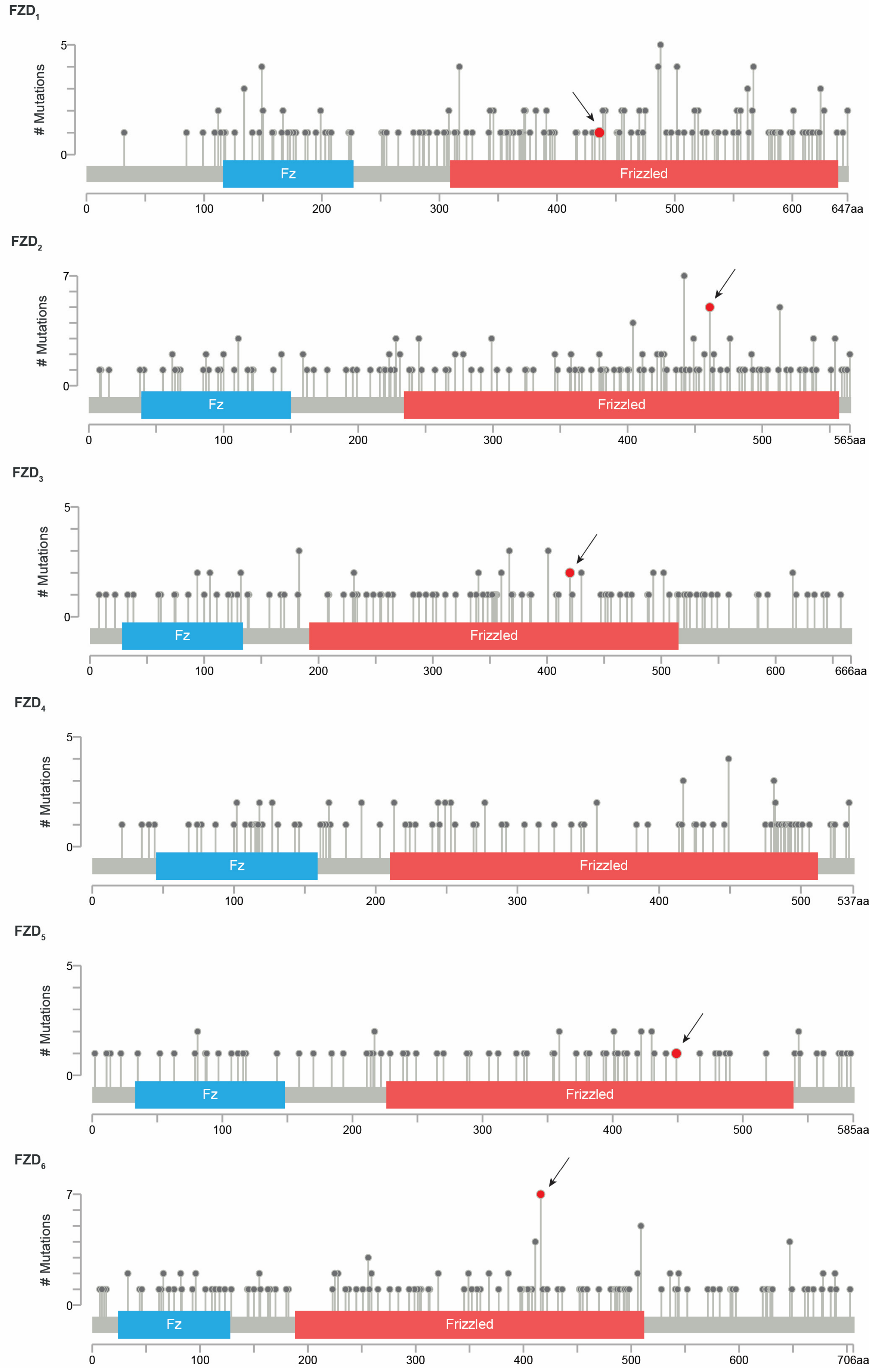

FZD1
Mutations
5
0
Fz
Frizzled
0
100
200
300
400
500
600
647aa
FZD2
Mutations
7
0
Fz
Frizzled
0
100
200
300
400
500
565aa
FZD3
Mutations
5
0
Fz
Frizzled
0
100
200
300
400
500
600
666aa
FZD4
Mutations
5
0
Fz
Frizzled
0
100
200
300
400
500
537aa
FZD5
Mutations
5
0
Fz
Frizzled
0
100
200
300
400
500
585aa
FZD6
Mutations
7
0
Fz
Frizzled
0
100
200
300
400
500
600
706aa

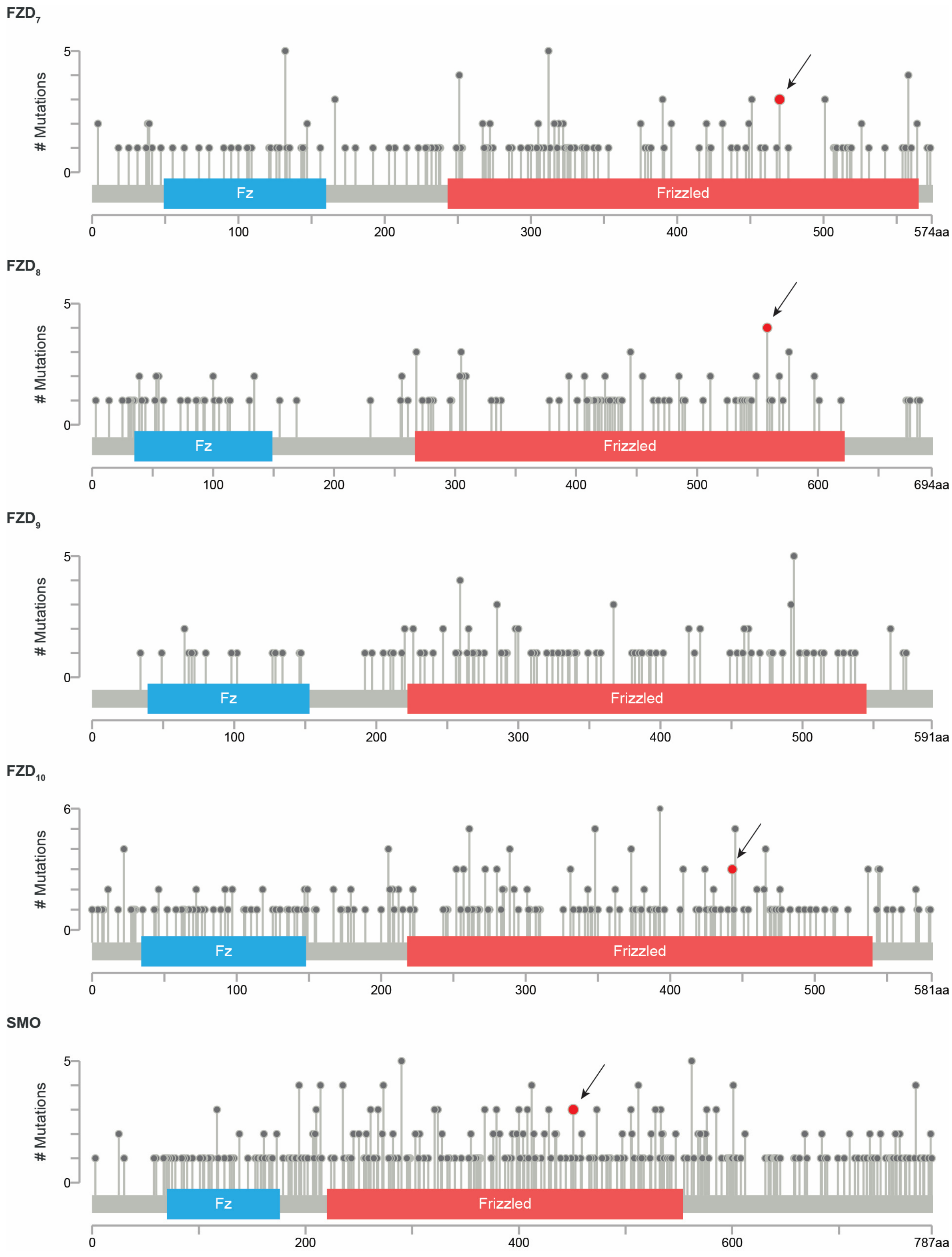

FZD7
Mutations
5
0
Fz
Frizzled
0
100
200
300
400
500
574aa
FZD8
Mutations
5
0
Fz
Frizzled
0
100
200
300
400
500
600
694aa
FZD9
Mutations
5
0
Fz
Frizzled
0
100
200
300
400
500
591aa
FZD10
Mutations
6
0
Fz
Frizzled
0
100
200
300
400
500
581aa
SMO
Mutations
5
0
Fz
Frizzled
0
200
400
600
787aa

b

Class F	Mutation in R/K$^{6.32}$	Associated tumor types	Mutation in W$^{7.55}$	Associated tumor types
FZD_1	R536H$^{6.32}$	Head and neck squamous cell carcinoma	None	None
FZD_2	R461H/C$^{6.32}$	Breast invasive ductal carcinoma, stomach adenocarcinoma, intestinal type stomach adenocarcinoma, mucinous adenocarcinoma of the colon and rectum, uterine endometrioid carcinoma	W538C$^{7.55}$	Uterine endometrioid carcinoma, pancreatic adenocarcinoma, cutaneous melanoma
FZD_3	R420Q$^{6.32}$	Colorectal adenocarcinoma	None	None
FZD_4	none	None	None	None
FZD_5	R449C$^{6.32}$	High-grade serous ovarian cancer	None	None
FZD_6	R416Q$^{6.32}$	Bladder urothelial, cervical squamous cell and uterine endometrioid carcinoma, colorectal adenocarcinoma	W493L$^{7.55}$	Prostate adenocarcinoma
FZD_7	R470H/C$^{6.32}$	Colorectal and prostate adenocarcinoma, uterine endometrioid carcinoma	None	None
FZD_8	R532C$^{6.32}$	Colorectal adenocarcinoma	None	None
FZD_9	none	None	None	None
FZD_{10}	R443H/C$^{6.32}$	Prostate and stomach adenocarcinoma, uterine endometrioid carcinoma, activated B cell type	None	None
SMO	R451S/H$^{6.32}$	Lung adenocarcinoma, uterine corpus endometrial carcinoma	W535L$^{7.55}$	Medulloblastoma, esophageal squamous cell carcinoma

Compiled from cBioportal for Cancer Genomics. Information is based on ca 68000 cancer cases.

Supplementary Figure 3. Mutational landscape in Class F receptors. (a) Lollipop graphs extracted from the cBioPortal for Cancer Genomics (*2, 3*) for all Class F receptors (FZD_{1-10} and SMO). The red arrow identifies the mutation in the R$^{6.32}$ residue present in all members of the family with the exception of FZD_4 and FZD_9, where there is a K$^{6.32}$ present. No mutations of the K$^{6.32}$ could be detected in the cBioPortal databases. Arrows mark FZD_1 R536H$^{6.32}$, FZD_2 R461H/C$^{6.32}$, FZD_3 R420Q$^{6.32}$, FZD_5 R449Q$^{6.32}$, FZD_6 R416Q$^{6.32}$, FZD_7 R470H/C$^{6.32}$, FZD_8 R532C$^{6.32}$, FZD_{10} R443H/C$^{6.32}$ and SMO R451S/H$^{6.32}$. No mutation was found in $FZD_{4,9}$ K$^{6.32}$. (b) Summarized data extracted from the cBioPortal for Cancer Genomics underlining mutations of the residues R/K$^{6.32}$ and W$^{7.55}$ in Class F receptors in diverse forms of cancer.

a

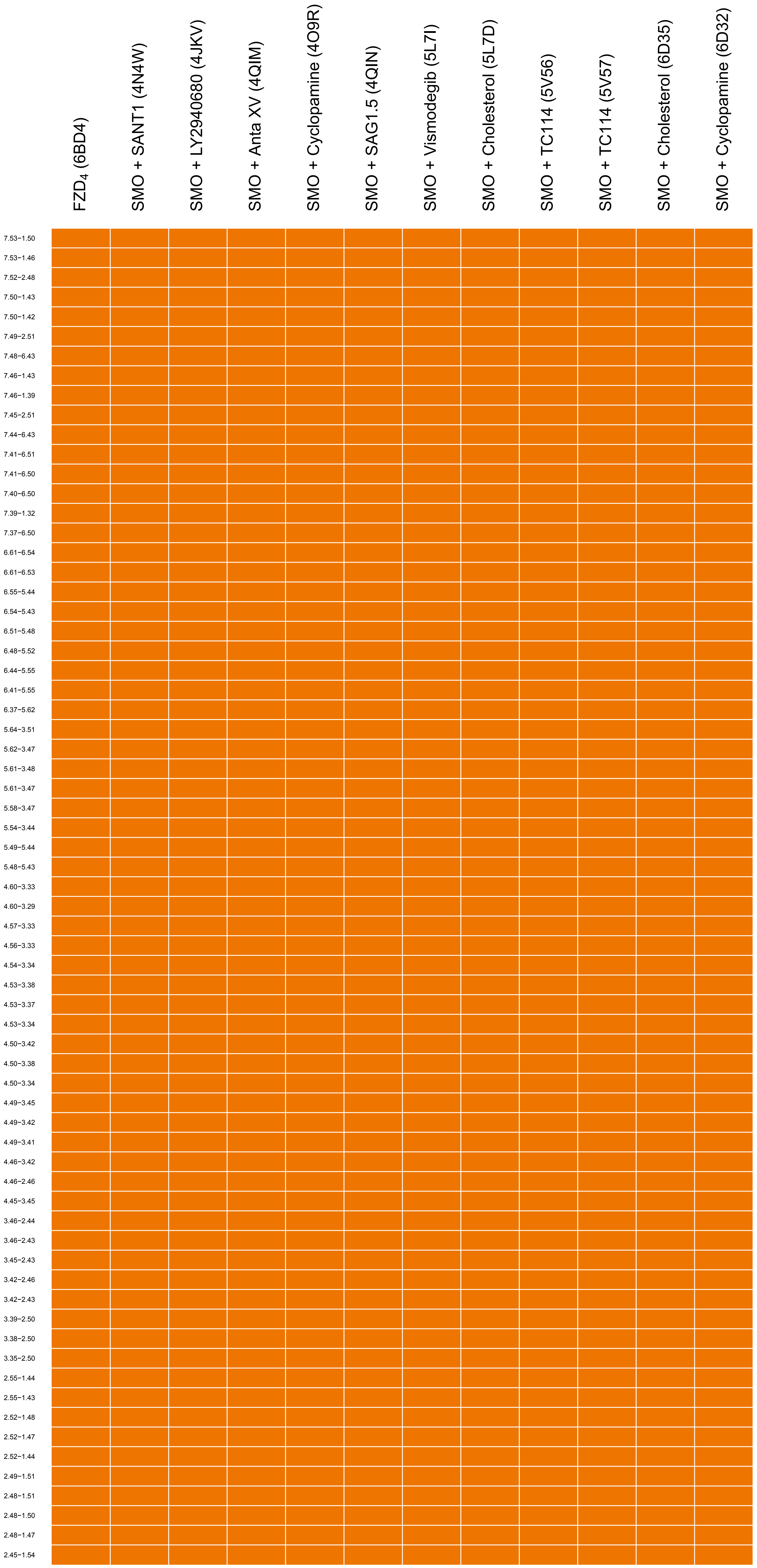
FZD$_4$ (6BD4)
SMO + SANT1 (4N4W)
SMO + LY2940680 (4JKV)
SMO + Anta XV (4QIM)
SMO + Cyclopamine (4O9R)
SMO + SAG1.5 (4QIN)
SMO + Vismodegib (5L7I)
SMO + Cholesterol (5L7D)
SMO + TC114 (5V56)
SMO + TC114 (5V57)
SMO + Cholesterol (6D35)
SMO + Cyclopamine (6D32)
7.53−1.50
7.53−1.46
7.52−2.48
7.50−1.43
7.50−1.42
7.49−2.51
7.48−6.43
7.46−1.43
7.46−1.39
7.45−2.51
7.44−6.43
7.41−6.51
7.41−6.50
7.40−6.50
7.39−1.32
7.37−6.50
6.61−6.54
6.61−6.53
6.55−5.44
6.54−5.43
6.51−5.48
6.48−5.52
6.44−5.55
6.41−5.55
6.37−5.62
5.64−3.51
5.62−3.47
5.61−3.48
5.61−3.47
5.58−3.47
5.54−3.44
5.49−5.44
5.48−5.43
4.60−3.33
4.60−3.29
4.57−3.33
4.56−3.33
4.54−3.34
4.53−3.38
4.53−3.37
4.53−3.34
4.50−3.42
4.50−3.38
4.50−3.34
4.49−3.45
4.49−3.42
4.49−3.41
4.46−3.42
4.46−2.46
4.45−3.45
3.46−2.44
3.46−2.43
3.45−2.43
3.42−2.46
3.42−2.43
3.39−2.50
3.38−2.50
3.35−2.50
2.55−1.44
2.55−1.43
2.52−1.48
2.52−1.47
2.52−1.44
2.49−1.51
2.48−1.51
2.48−1.50
2.48−1.47
2.45−1.54

b

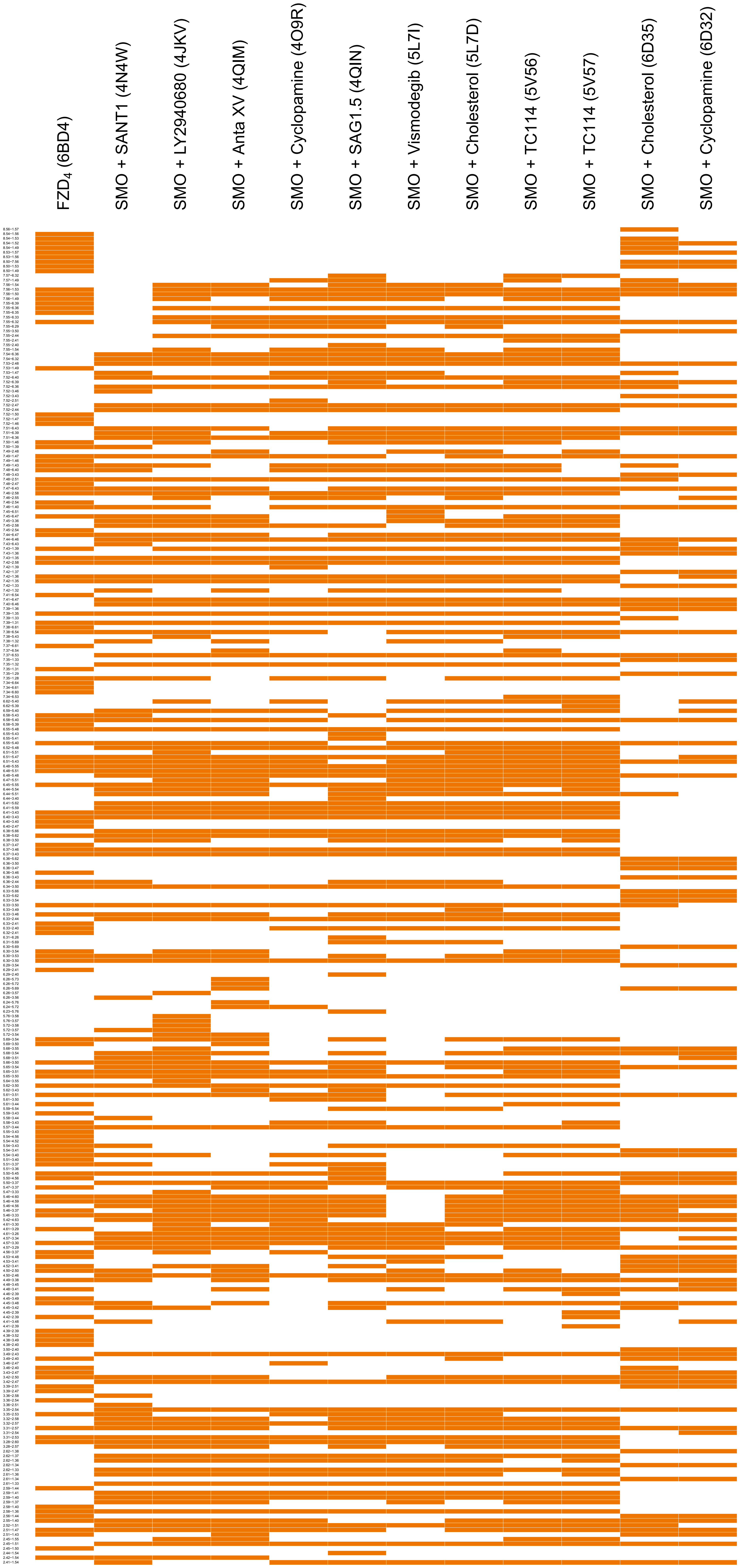
FZD4 (6BD4)
SMO + SANT1 (4N4W)
SMO + LY2940680 (4JKV)
SMO + Anta XV (4QIM)
SMO + Cyclopamine (4O9R)
SMO + SAG1.5 (4QIN)
SMO + Vismodegib (5L7I)
SMO + Cholesterol (5L7D)
SMO + TC114 (5V56)
SMO + TC114 (5V57)
SMO + Cholesterol (6D35)
SMO + Cyclopamine (6D32)

Supplementary Figure 4. Smoothened receptor clustering according to interhelical contact fingerprints. Analyzed structures included smoothened receptor bound to the antagonists SANT1 (PDB ID: 4N4W), Anta XV (PDB ID: 4QIM), LY2940680 (PDB ID: 4JKV), vismodegib (PDB ID: 5L7I), TC114 (PDB IDs: 5V56; 5V57), a cholesterol molecule in the receptor extracellular cysteine-rich domain (PDB ID: 5L7D; 6D35), cyclopamine (PDB ID: 4O9R), the agonist SAG1.5 (PDB ID: 4QIN), two molecules of cyclopamine bound to the CRD and the 7TM core (PDB ID: 6D32) (an orange box indicates that the contact is present in that structure, a white box indicates absence of the contact). (a) Shared interhelical contacts across all structures. (b) Unshared interhelical contacts across all structures.

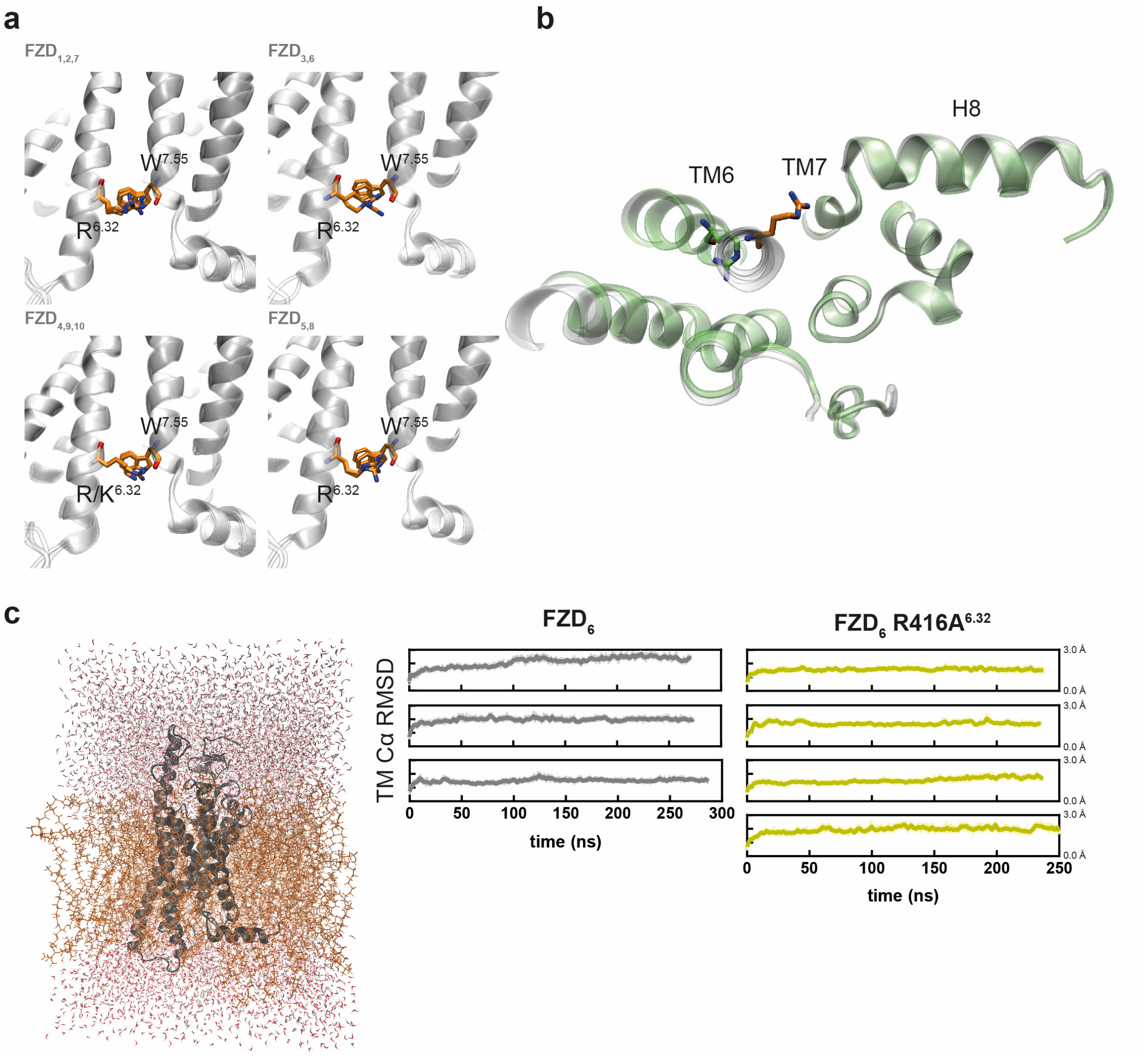

Supplementary Figure 5. Modelling the molecular switch region in Class F receptors. (a) Presentation of interactions between R/K$^{6.32}$ in TM6 and W$^{7.55}$ in TM7 for FZD$_{1\text{-}10}$ subdivided into homology clusters as in Fig. 5. Models of FZDs are based on a monomer of the SMO structure provided in the PBD ID: 4JKV. (b) Alignment of inactive (white) and active-like (green) FZD$_6$ models showing the outward displacement of lower end of TM6 in the active-like state with R416$^{6.32}$ represented as sticks. (c) Representation of the membrane-inserted and equilibrated FZD$_6$ model; protein is shown as grey cartoon, lipids and water molecules are represented as orange and white-red sticks respectively. The RMSD plots (carbon Cα atoms of the transmembrane domains of FZD$_6$ and FZD$_6$ R416A$^{6.32}$) of MD simulations are shown. Thick traces indicate the moving average smoothed over a 2 ns window and thin traces represent raw data.

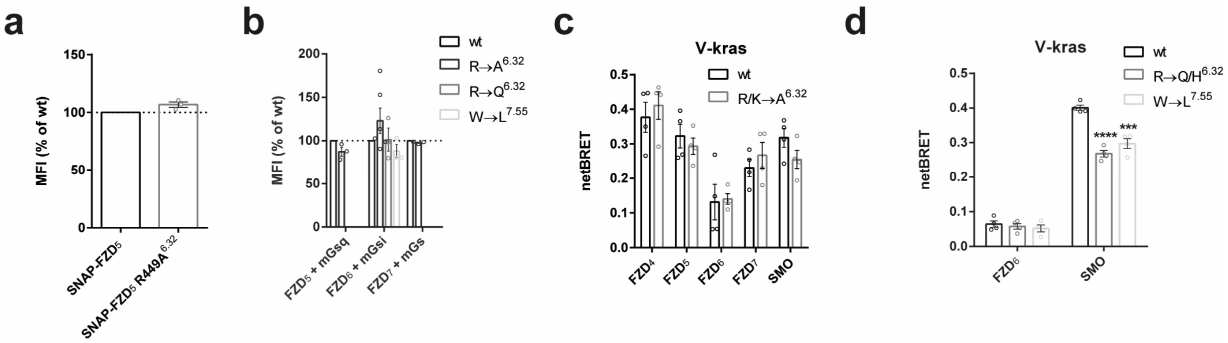

Supplementary Figure 6. Surface expression of overexpressed Class F receptors. (a-c) All SNAP-tagged receptors were assessed for surface expression by flow cytometry under their respective experimental conditions. (a) FZD_5 wt and R449A$^{6.32}$ were assessed by flow cytometry for TOPFlash experiments (two-tailed *t*-test). (b) wt, R/K$^{6.32}$ and W$^{7.55}$ mutants for FZD_5, FZD_6 and FZD_7 were assessed by flow cytometry in combination with mG proteins. Data are represented as mean ± s.e.m. of n=3 independent experiments, no statistically significant differences were found between surface expression levels of wt and R/K$^{6.32}$ mutants of each receptor (one-way ANOVA). (c,d) All wt and K/R$^{6.32}$ mutant Class F receptor constructs were assessed for subcellular localization using bystander BRET between receptor (donor) and Venus-tagged acceptor molecules directed to the plasma membrane (V-kras). Constructs were coexpressed in HEK293 cells and the BRET ratio between Venus and *R*luc8/Nluc was measured. White 96 well plates were used in c and black ones were used in d. $F(2,9)=41.63$. *** $P < 0.001$, **** $P < 0.0001$ (one-way ANOVA). Gating parameters for flow cytometry can be seen in Supplementary Fig. 10.

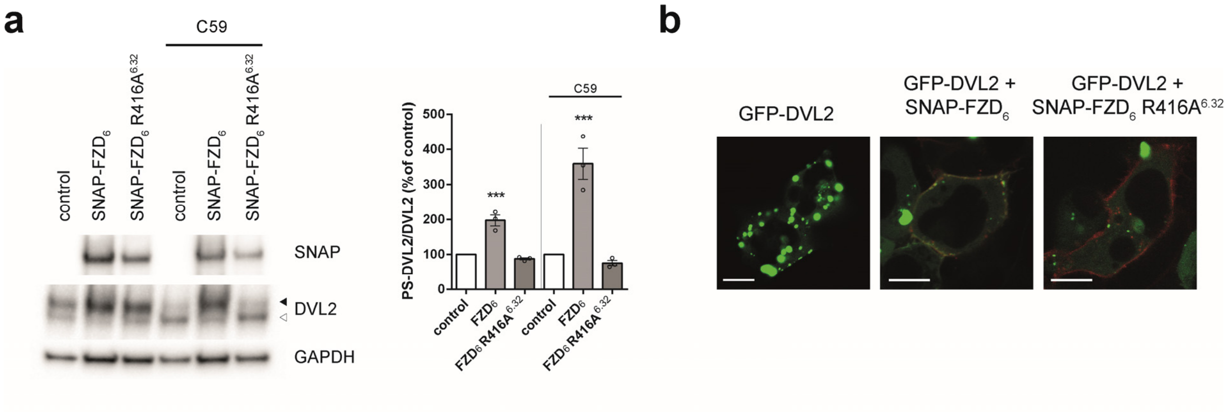

Supplementary Figure 7. The effect of wt and R416A$^{6.32}$ FZD$_6$ on the electrophoretic mobility and subcellular localization of DVL. (a) HEK293 cells stably transfected with SNAP-FZD$_6$, SNAP-FZD$_6$ R416A$^{6.32}$ and control cells were lyzed and analyzed by immunoblotting using anti-SNAP, -DVL2 and -GAPDH antibodies. The formation of endogenous PS-DVL2 (phosphorylated and shifted DVL) was monitored similar to what we have done before[3]. Experiments were performed in the absence and presence of 5 nM of the Porcupine inhibitor C59 (overnight treatment). Data are represented as mean ± s.e.m. of n=4 independent experiments; $P=0.0003$, $F(2,6)=42.21$ (untreated with C59); $P=0.0004$, $F(2,6)=37.02$ (treated with C59). *** $P < 0.001$ (one-way ANOVA). (b) Representative images of HEK293 cells transfected with GFP-DVL2 alone or in combination with SNAP-FZD$_6$ or SNAP-FZD$_6$ R416A$^{6.32}$. Note that the typical punctate appearance of GFP-DVL2 distribution was altered to a mostly membranous localization when SNAP-FZD$_6$ was present, but not when SNAP-FZD$_6$-R416A$^{6.32}$ was coexpressed. Size bar = 10 μm. Uncropped immunoblots for (a) are found in Supplementary Fig. 11.

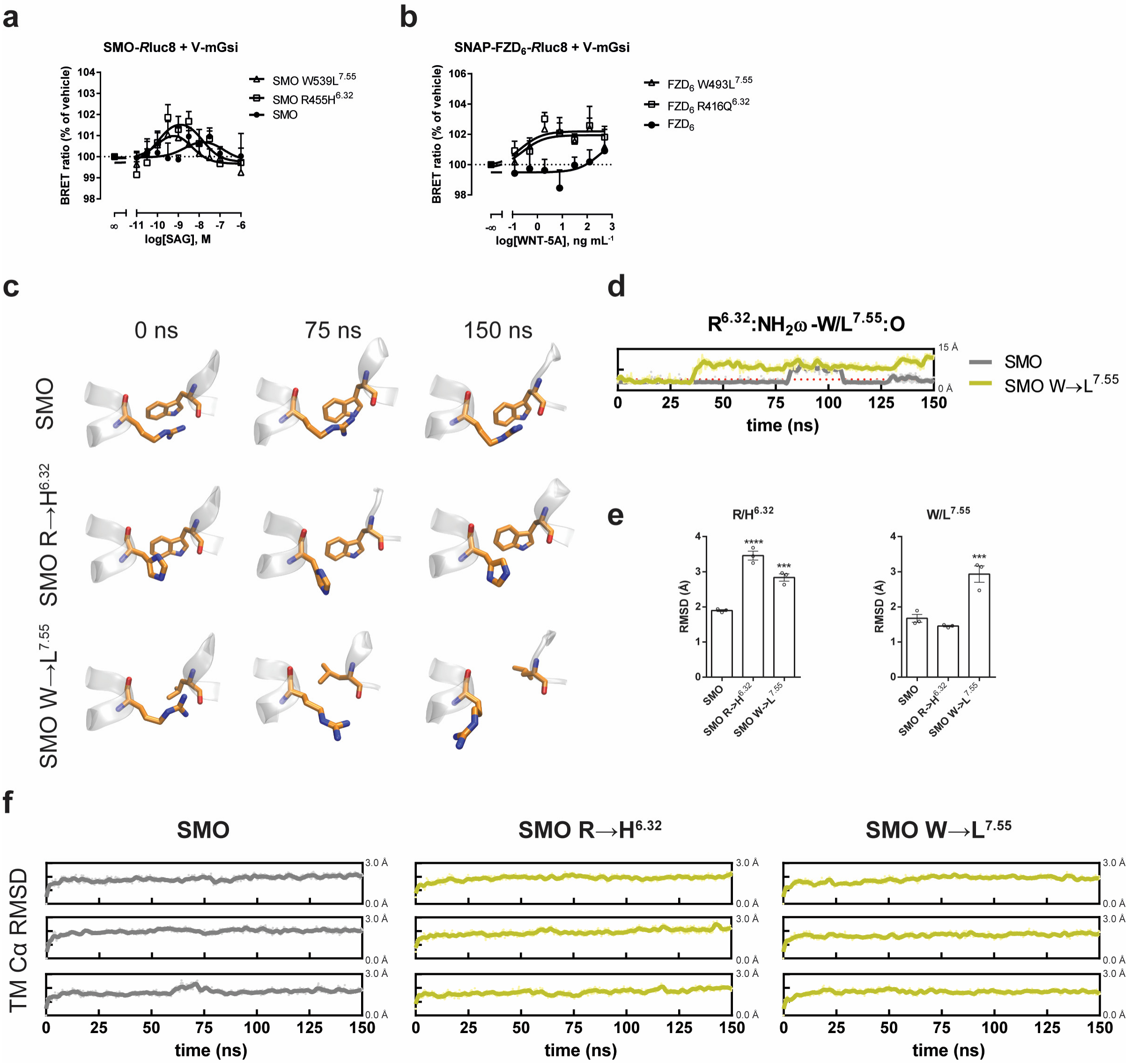

Supplementary Figure 8. Naturally occurring cancer mutations in the molecular switch of SMO and FZD₆ mimic the phenotype of the experimental alanine mutants. BRET experiments in HEK293 cells transiently expressing: (a) mouse SMO (n=7; closed circle), SMO R455H$^{6.32}$ (n=8; open square), SMO W539L$^{7.55}$ (n=8; open triangle) with mGsi; (b) FZD$_6$ (n=4; closed circle), FZD$_6$ R416Q$^{6.32}$ (n=4; open square), FZD$_6$ W493L$^{7.55}$ (n=4; open triangle) with mGsi. Data are represented as mean ± s.e.m. In contrast to the data shown in Fig. 5, experiments were performed in black 96 well plates. (c) Representative snapshots of MD simulations for human SMO, SMO R→H$^{6.32}$, SMO W→L$^{7.55}$ at time point 0 ns, 75 ns and 150 ns. Simulations were restrained for 50 ns to equilibrate the system prior to release at the time point 0 ns. (d) Representative distance plots of three independent MD simulations (150 ns each) of the NH$_2$ω nitrogen of R$^{6.32}$ and the oxygen of the W$^{7.55}$ in human SMO and SMO W→L$^{7.55}$. The dotted line (red) indicates the maximum distance (4 Å) that is still likely to allow polar interactions. (e) Bar graphs provide average RMSD ± s.e.m. over simulation time (150 ns) of the heavy atoms for the individual residues R/H$^{6.32}$ and W/L$^{7.55}$ for human SMO, SMO R→H$^{6.32}$, SMO W→L$^{7.55}$. $F(2,6)=68.85$ (R→H$^{6.32}$); $P=0.0009$, $F(2,6)=28.66$ (W→L$^{7.55}$).*** $P < 0.001$, **** $P < 0.0001$ (one-way ANOVA). (f) The RMSD plots (carbon Cα atoms of the transmembrane domains of human SMO, SMO R→H$^{6.32}$, SMO W→L$^{7.55}$) of three independent MD simulations are shown. Thick traces indicate the moving average smoothed over a 2 ns window and thin traces represent raw data.

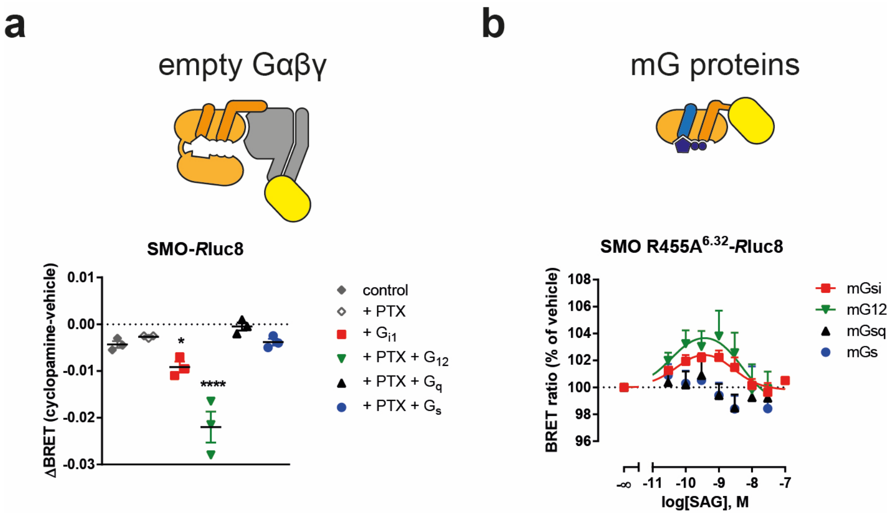

Supplementary Figure 9. SMO R455A$^{6.32}$ maintains its G protein coupling profile. (a) Recruitment of empty heterotrimers to SMO. Gs/q/12-KO HEK293 cells were transiently transfected with luciferase-tagged SMO, untagged Gα (G_{i1} – red square; G_{12} – green triangle; G_q – black triangle; G_s – blue circle) or pcDNA3.1 (control; grey, filled diamond) and split Gβγ-V. In order to inhibit endogenous $G_{i/o}$ activity, cells were co-transfected with the S1 subunit of pertussis toxin (PTX; PTX with pcDNA control – grey open diamond). Cells were permeabilized with digitonin (1 μg ml^{-1}) and incubated with the inverse agonist cyclopamine (10 μM) or vehicle and apyrase (2 units ml^{-1}). The difference between apyrase/vehicle (constitutive coupling) and apyrase/cyclopamine is represented by the difference in BRET (ΔBRET). Data are represented as mean ± s.e.m. of n=3 independent experiments; $F(6,14)=25.72$.* $P < 0.05$, **** $P < 0.0001$ (one-way ANOVA). (b) Agonist-induced recruitment of mG proteins to SMO R455A$^{6.32}$. SMO R455A$^{6.32}$ and representative mG proteins of four Gα subclasses (mGsi – red square; mG12 – green triangle; mGsq – black triangle; mGs – blue circle) were co-transfected in HEK293 cells and the agonist (SAG)-induced coupling profile of the mutant receptor was assessed by BRET between SMO-*R*luc8 and the mGsi, mGs, mGsq or mG12. Data are represented as mean ± s.e.m. of n=5-7 independent experiments.

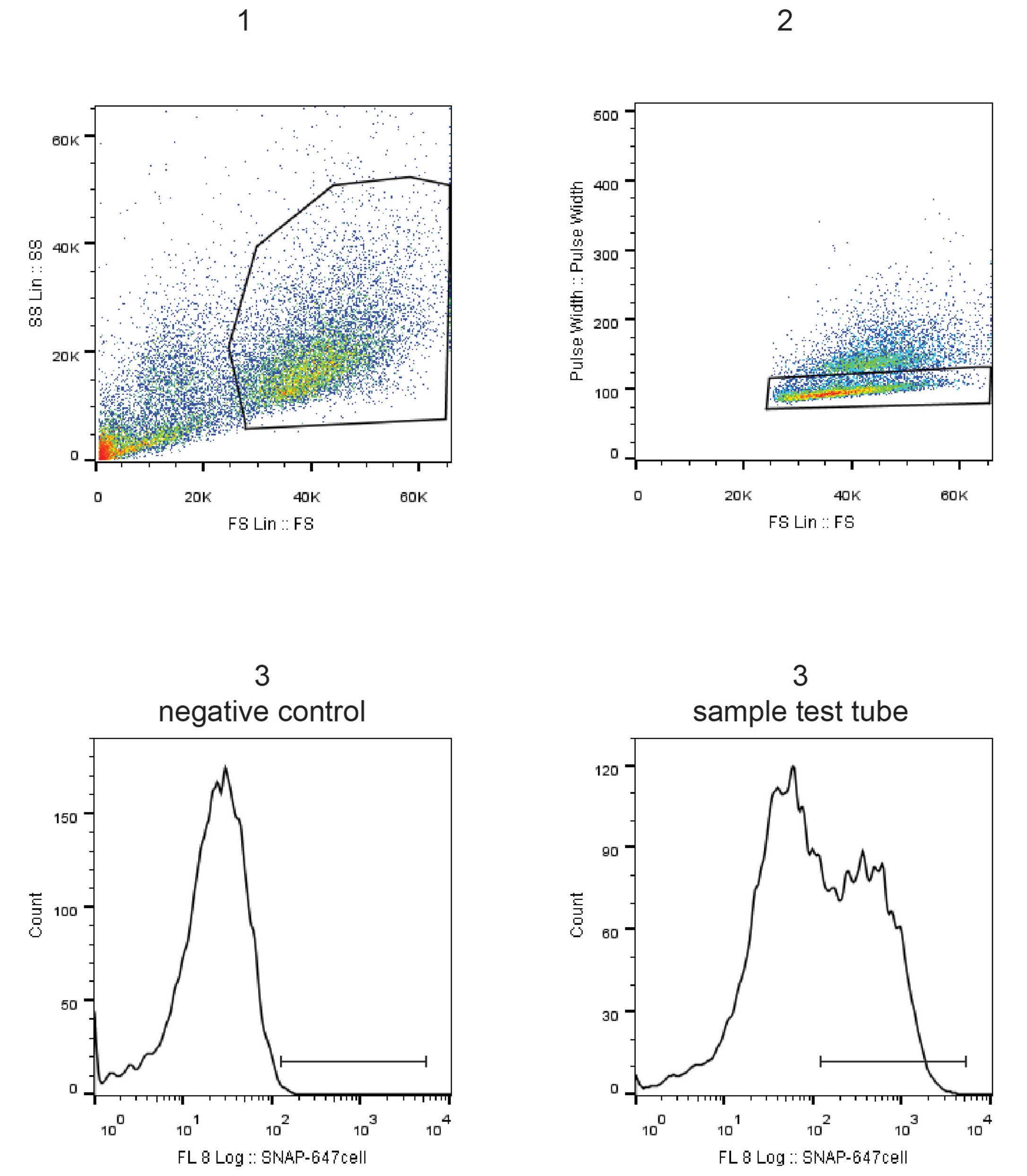

Supplementary Figure 10. Gating parameters for flow cytometry. Cells were gated using the FS/SS plot to exclude dead cells and cell debris as shown in (1). The doublets were excluded based on the FS/pulse width plot as shown in (2). Background fluorescence was defined using mock control/control cells (3).

Uncropped immunoblots for Supplementary Fig. 7a

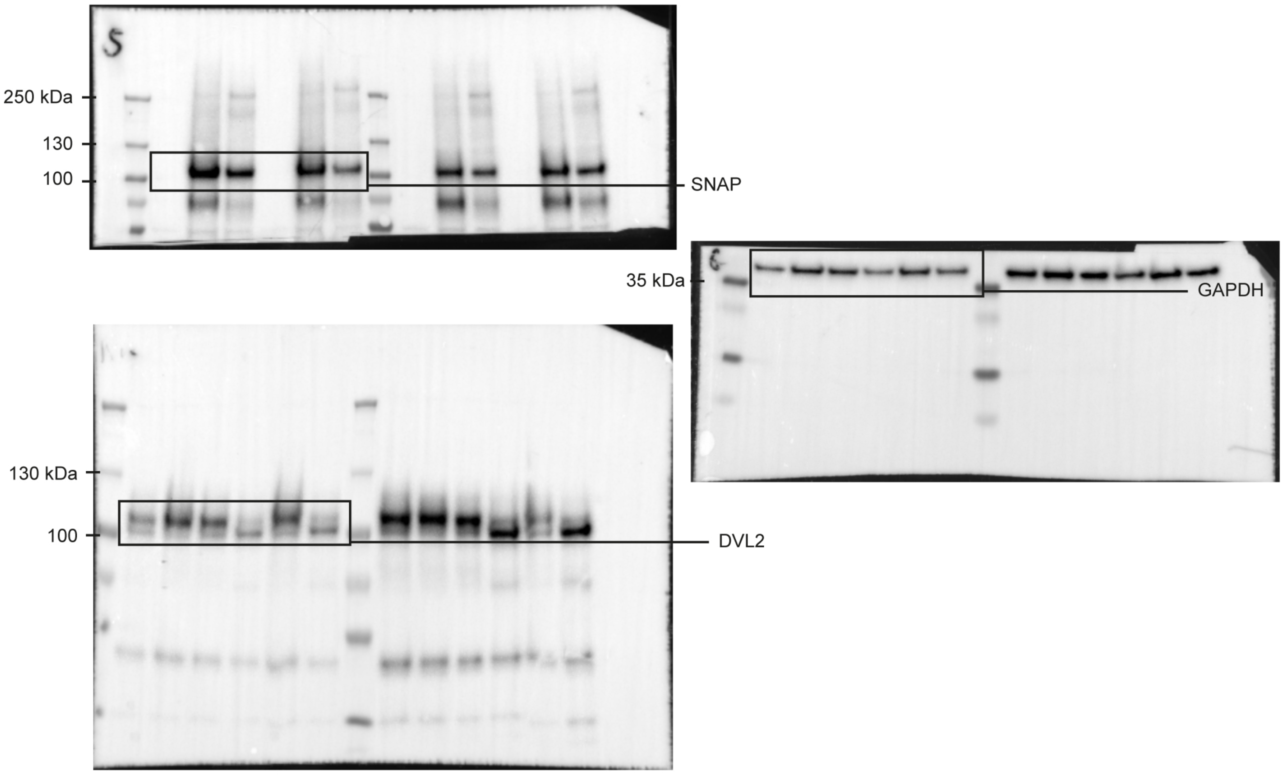

Uncropped immunoblots for Fig. 4b

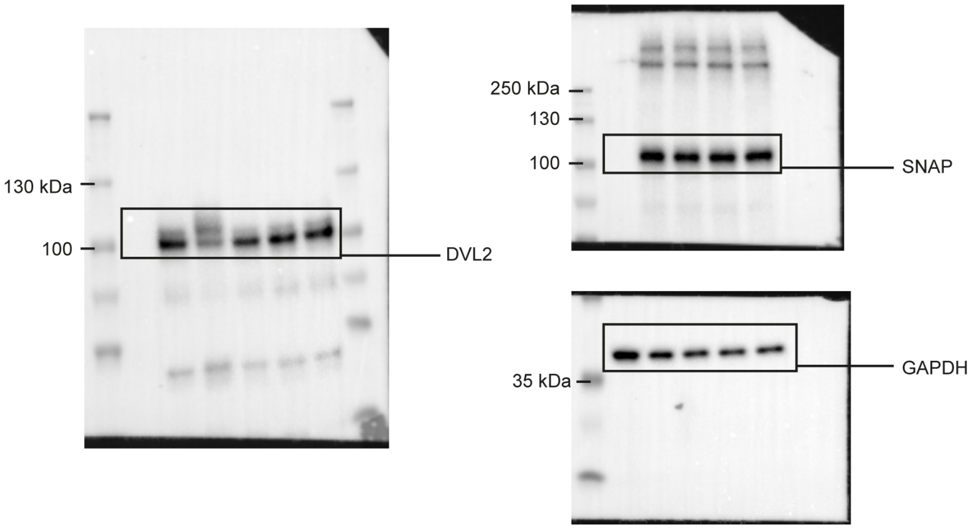

Uncropped immunoblots for Fig. 4d

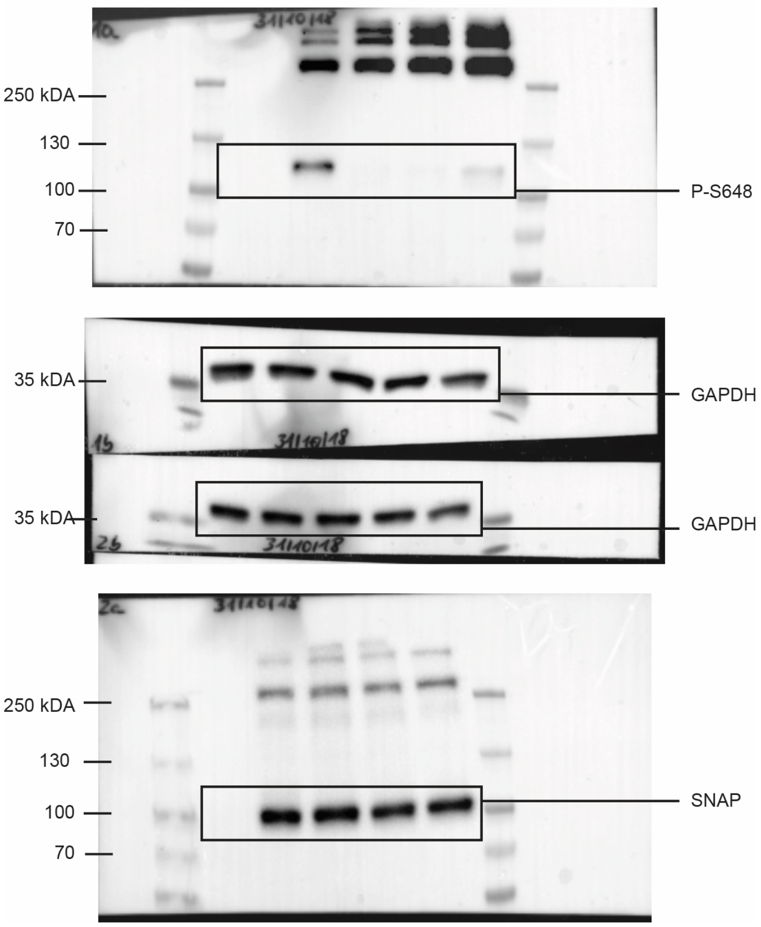

Supplementary Figure 11. Uncropped immunoblots. Immunoblots for SNAP and GAPDH were run on the same 4-20% gel. In order to quantify the ratio of PS-DVL2/DVL2, samples were run in parallel with the same loading on a 7.5% gel.

References

1. O. Wagih, ggseqlogo: a versatile R package for drawing sequence logos. *Bioinformatics* **33**, 3645-3647 (2017).
2. J. Gao *et al.*, Integrative analysis of complex cancer genomics and clinical profiles using the cBioPortal. *Sci Signal* **6**, pl1 (2013).
3. E. Cerami *et al.*, The cBio cancer genomics portal: an open platform for exploring multidimensional cancer genomics data. *Cancer discovery* **2**, 401-404 (2012).
