# Supplementary material for: A conserved molecular switch in Class F receptors regulates receptor activation and pathway selection
Source: Nat Commun. 2019 Feb 8;10:667. doi: 10.1038/s41467-019-08630-2 (PMC6368630; doi:10.1038/s41467-019-08630-2)
Supplement: Supplementary file 3 — Reporting Summary [file 41467_2019_8630_MOESM3_ESM.pdf]

nature research

Corresponding author(s): Gunnar Schulte

Reporting Summary

Nature Research wishes to improve the reproducibility of the work that we publish. This form provides structure for consistency and transparency in reporting. For further information on Nature Research policies, see Authors & Referees and the Editorial Policy Checklist.

Statistical parameters

When statistical analyses are reported, confirm that the following items are present in the relevant location (e.g. figure legend, table legend, main text, or Methods section).

n/a	Confirmed	
☐	☒	The exact sample size (*n*) for each experimental group/condition, given as a discrete number and unit of measurement
☐	☒	An indication of whether measurements were taken from distinct samples or whether the same sample was measured repeatedly
☐	☒	The statistical test(s) used AND whether they are one- or two-sided<br>*Only common tests should be described solely by name; describe more complex techniques in the Methods section.*
☐	☒	A description of all covariates tested
☐	☒	A description of any assumptions or corrections, such as tests of normality and adjustment for multiple comparisons
☐	☒	A full description of the statistics including central tendency (e.g. means) or other basic estimates (e.g. regression coefficient) AND variation (e.g. standard deviation) or associated estimates of uncertainty (e.g. confidence intervals)
☐	☒	For null hypothesis testing, the test statistic (e.g. *F*, *t*, *r*) with confidence intervals, effect sizes, degrees of freedom and *P* value noted<br>*Give P values as exact values whenever suitable.*
☒	☐	For Bayesian analysis, information on the choice of priors and Markov chain Monte Carlo settings
☒	☐	For hierarchical and complex designs, identification of the appropriate level for tests and full reporting of outcomes
☒	☐	Estimates of effect sizes (e.g. Cohen's *d*, Pearson's *r*), indicating how they were calculated
☐	☒	Clearly defined error bars<br>*State explicitly what error bars represent (e.g. SD, SE, CI)*

Our web collection on statistics for biologists may be useful.

Software and code

Policy information about availability of computer code

Data collection	Reader Control Software and Mars for BMG CLARIOstar plate reader, Summit for ADP Cyan flow cytometer, Gen5 for Synergy 2 plate reader, ZEISS ZEN for ZEISS LSM510 confocal microscope, Image Lab for BioRad ChemiDoc, NAMD and GROMACS for molecular dynamics, Wallac for EnVision plate reader.
Data analysis	GraphPad Prism 5 or 6, FlowJo V10, VMD 1.9.x, PyMol 2.0, MS Excel 2007 or 2013, Image Lab, ImageJ.

For manuscripts utilizing custom algorithms or software that are central to the research but not yet described in published literature, software must be made available to editors/reviewers upon request. We strongly encourage code deposition in a community repository (e.g. GitHub). See the Nature Research guidelines for submitting code & software for further information.

Data

Policy information about availability of data

All manuscripts must include a data availability statement. This statement should provide the following information, where applicable:

- Accession codes, unique identifiers, or web links for publicly available datasets
- A list of figures that have associated raw data
- A description of any restrictions on data availability

The data that support the findings of this study are presented within the article, its Extended Data and Supplementary Data files and are available from the

corresponding author upon reasonable request. All constructs originally described in this study can be obtained and used under a material transfer agreement for non-commercial purposes on request from the corresponding author.

Field-specific reporting

Please select the best fit for your research. If you are not sure, read the appropriate sections before making your selection.

☒ Life sciences ☐ Behavioural & social sciences ☐ Ecological, evolutionary & environmental sciences

For a reference copy of the document with all sections, see nature.com/authors/policies/ReportingSummary-flat.pdf

Life sciences study design

All studies must disclose on these points even when the disclosure is negative.

Sample size	Described in figure legends
Data exclusions	N/A
Replication	N/A
Randomization	N/A
Blinding	N/A

Reporting for specific materials, systems and methods

Materials & experimental systems

n/a	Involved in the study	
☒	☐	Unique biological materials
☐	☒	Antibodies
☐	☒	Eukaryotic cell lines
☒	☐	Palaeontology
☒	☐	Animals and other organisms
☒	☐	Human research participants

Methods

n/a	Involved in the study	
☒	☐	ChIP-seq
☐	☒	Flow cytometry
☒	☐	MRI-based neuroimaging

Antibodies

Antibodies used	All the catalog numbers are provided in the Materials section. The anti-P-S648-FZD6 was custom made.
Validation	Validation of the P-S648 FZD6 antibody was previously published in doi: 10.1074/jbc.RA118.004656 (Strakova K et al 2018, J Biol Chem)

Eukaryotic cell lines

Policy information about cell lines

Cell line source(s)	Yes
Authentication	No
Mycoplasma contamination	Yes, it was regularly checked. See : Methods section / Cell culture paragraph
Commonly misidentified lines (See ICLAC register)	Yes

Flow Cytometry

Plots

Confirm that:

☒ The axis labels state the marker and fluorochrome used (e.g. CD4-FITC).

☒ The axis scales are clearly visible. Include numbers along axes only for bottom left plot of group (a 'group' is an analysis of identical markers).

☒ All plots are contour plots with outliers or pseudocolor plots.

☒ A numerical value for number of cells or percentage (with statistics) is provided.

Methodology

Sample preparation	see method section, paragraph "Flow cytometry"
Instrument	ADP Cyan flow cytometer
Software	FlowJo V10 (Tree Star)
Cell population abundance	see method section, paragraph "Flow cytometry" and Supplementary figure 6a-c. Surface expression of overexpressed Class F receptors. At least 10,000 events were collected.
Gating strategy	see Supplementary figure 10. Gating parameters for flow cytometry.

☒ Tick this box to confirm that a figure exemplifying the gating strategy is provided in the Supplementary Information.
