# Supplementary material for: A conserved molecular switch in Class F receptors regulates receptor activation and pathway selection
Source: Nat Commun. 2019 Feb 8;10:667. doi: 10.1038/s41467-019-08630-2 (PMC6368630; doi:10.1038/s41467-019-08630-2)
Supplement: Supplementary file 4 — Description of Additional Supplementary Files [file 41467_2019_8630_MOESM4_ESM.docx]

**Title:** Supplementary Data 1 (Class F mega alignment)
**Description:** Multiple sequence alignment of Class F homologs. Sequences for one-to-one orthologs for each Class F receptor in human were downloaded from Ensembl for all species with the exception of S. cerevisiae using the BiomaRt package. Orthologs with homology confidence 1 were retained and corresponding sequences were aligned using MAFFT in the G-INS-i mode.
